# Hot Pepper (*Capsicum annuum* L.) Fusarium Wilt (*Fusarium oxysporum* f. sp. *capsici*) Distribution, Epidemiology, and Management Methods in Ethiopia

**DOI:** 10.1155/tswj/3010436

**Published:** 2026-08-13

**Authors:** Ermias Misganaw Amare

**Affiliations:** ^1^ College of Agriculture and Environment Science, Department of Horticulture, University of Gondar, Gondar, Ethiopia, uog.edu.et

**Keywords:** *Fusarium oxysporum* f. sp. *capsici*, Fusarium wilt, IPM, pepper

## Abstract

Hot pepper (*Capsicum annuum* L.) is an important cash crop and dietary staple in Ethiopia, providing essential income for smallholder farmers and vital nutrients. However, pepper production is severely constrained by a devastating soil‐borne fungal disease, *Fusarium oxysporum* f. sp. *capsici.* It is the fifth most economically important fungal phytopathogen. In Ethiopia, wilt‐causing fungal pathogens were reported to be the leading problem of pepper crops, causing huge yield losses. Warm and humid conditions prevalent in major pepper‐growing regions, such as Amhara, Oromia, and the South, are highly conducive to the disease. The pathogen′s longevity in the soil and ability to spread via infected seeds, seedlings, tools, and irrigation water make it a persistent threat. The pathogen has shown a significant variation in disease severity across and within the major pepper‐growing regions. In the Amhara region, a severity of 70% in Jabi Tehena district and a severity of 15% in Guagusa Shekudad district were documented. Similarly, in the southern region, pepper Fusarium wilt disease incidence reached 91% in Alaba district and 79% in Meskan district. In the Oromia region, incidence was recorded at 50% in Dugda district and 19% in Adami Tullu district. In the Assosa and Kamashi Zones, it reached up to 50%. These observed variations in pepper Fusarium wilt disease severity are attributed to various biophysical factors such as agroecological differences, cropping systems, seed sources, disease management methods, and agronomic practices. These disease‐causing factors highlight the need to develop holistic disease management practices. *F. oxysporum* management is challenging due to the pathogen′s genetic diversity, limited research activity, and poor extension services. Therefore, this review is aimed at synthesizing the current status, pathogen ecology, epidemiology, and management strategies of pepper Fusarium wilt disease in Ethiopia.

## 1. Introduction

Hot pepper (*Capsicum annuum* L.) belongs to the Solanaceae family and the genus *Capsicum* [1]. It evolved in the New World tropics and subtropics (Mexico, Central America, and Andes of South America) over 2000 years ago [2]. The genus *Capsicum* consists of approximately 22 wild and 5 domesticated species. Among the cultivated species, *C. annuum* is the most widely distributed worldwide [3]. Hot pepper is a prominent cash crop for smallholder farmers in many developing countries, such as Bhutan, Cambodia, China, Ethiopia, Ghana, India, Indonesia, Nigeria, Pakistan, and Thailand [4]. Pepper is one of the most important spice crops widely cultivated for its pungent flavor and aroma [5]. Its production requires a hot, dry climate, and a frost‐free agroecological environment. In Ethiopia, the suitable conditions for optimal production of pepper are an altitude range between 1000 and 1800 masl, a mean annual rainfall of 600–1200 mm, and a mean annual temperature of 25°C–28°C [6].

Globally, hot pepper is the most economically significant vegetable crop and is consumed as fresh, dried, or processed into spices and condiments. In Ethiopia, hot pepper is the most commonly used spice in the daily diet of the society. The average daily consumption of hot pepper by Ethiopian adults is estimated to be 15 g, which is higher than that of most other vegetables [7]. Pepper is also an important crop for spice extraction as it contains oleoresin, and Ethiopia is among the few developing countries that have been producing paprika and *Capsicum* oleoresin for export markets [8]. Amhara, Oromia, and South Nation and Nationality (SNNP) are the leading hot pepper‐producing regions in the country [9]. Hot pepper production is a feasible enterprise that can increase income, reduce poverty, create jobs, and improve the food security of all households [10]. In recent times, the price of hot pepper in major Ethiopian marketplaces has jumped from 2 USD (2022) to 5 USD per kg (since 2024). During the 2020/2021 Meher cropping season, the total cultivated area of pepper (red and green) was 170,093.66 ha, and the total production was 301,148.562 t [6]. Despite hot pepper′s economic significance, its production faces constraints due to abiotic and biotic factors. In Ethiopia, the major production bottlenecks of pepper crop are the use of local varieties, poor agronomic practices, and the increasing prevalence and intensity of fungal, bacterial, and viral diseases [11]. According to MoARD [12], the main diseases that directly cause low yields in pepper are pepper mottle virus, and fungal diseases including damping off (*Rhizoctonia solani*, *Pythium* spp., and *Fusarium* spp.), powdery mildew, blight (*Phytophthora capsici*), and anthracnose (*Colletotrichum capsici*). According to Shiferaw and Alemayehu [13], fungal (Fusarium wilt and powdery mildew), bacterial (leaf spot and soft rot), and viral diseases are the most frequently encountered diseases in hot pepper‐producing areas of southern Ethiopia. In Ethiopia, Fusarium wilt has emerged as a serious problem in recent years [14, 15]. According to Teshome [16], in Ethiopia, Bako areas, Fusarium wilt resulted in a 68%–70% yield loss. In the central rift valley of Ethiopia, Fusarium wilt disease incidence was 15%–47% [14]. In Ethiopia, major pepper‐growing areas consistently reported high levels of Fusarium wilt disease and severe yield loss as the pathogen has become established due to continuous cultivation with monocropping. Research results found that Fusarium wilt is one of the most frequently occurring disease of pepper in NW Ethiopia [15, 17]. *Fusarium oxysporum* f. sp. *capsici* is the most important causative agent of severe vascular wilts and root rot diseases in hot pepper and ultimately brings heavy economic losses. In Ethiopia, wilt‐causing fungal pathogens were reported to be the leading problem of pepper crops, causing 86% or above yield losses [18]. Fulya Baysal‐Gurel and Kabir [19] found that pathogens such as *Rhizoctonia* spp., *Fusarium* spp., *Verticillium* spp., *Pythium* spp., *and Phytophthora* spp. can lead to yield losses of 50%–75% in crops like vegetables, fruits, and ornamentals. The objectives of this review are to synthesize the current status, pathogens′ biology, epidemiology, and management strategies of pepper Fusarium wilt disease in Ethiopia.

## 2. Hot Pepper Production Status in Ethiopia

**Figure 1:**
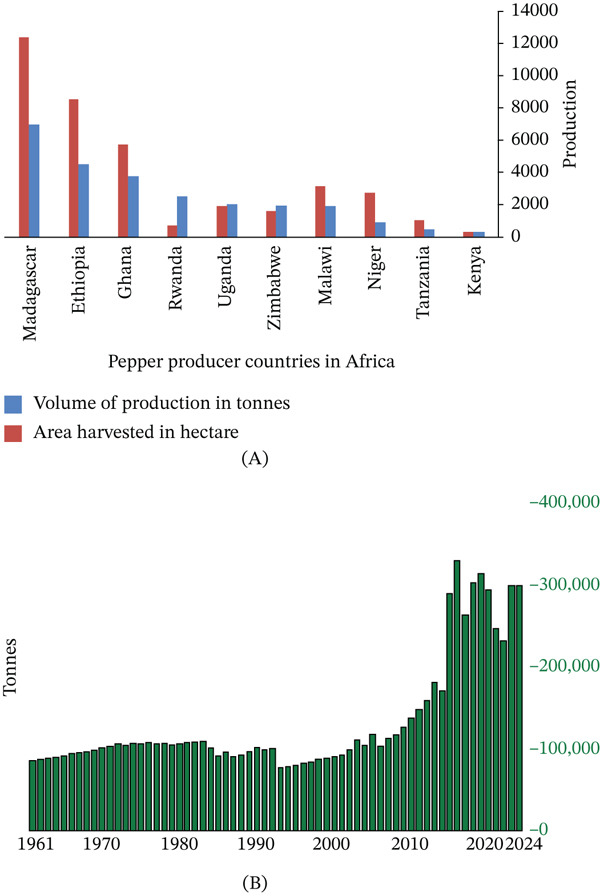
(A) Top 10 pepper producer countries in Africa; source: FAOSTAT [21]. (B) Chili and pepper (dry) production in Ethiopia from 1961 to 2024; source: Helgi Library.

In Ethiopia, pepper is cultivated in several parts of the country in different agroecological conditions. The production of pepper was 249,289 t with a productivity of 1.6 t/ha from 157,657 ha of land in the 2021/2022 main cropping season [20]. In 2021, Ethiopia was one of the top 10 producers of dry pepper in Africa (Figure 1A). Nigeria and Ethiopia are the largest producers of both green and dry pepper in the region (Figure 1B). In Eastern and Southern African countries, particularly Ethiopia and Uganda, a diverse range of pepper cultivars is cultivated based on their local climatic conditions and market demands [22]. The most common pepper cultivars in these regions belong to the *C. annuum* species [23]. In Ethiopia, *C. annuum* is extensively grown, and popular cultivars include “Markofana” and “Melka shote” [24]. According to Solomon et al. [25], in Ethiopia, out of the nationwide collection of pepper germplasm, 132 accessions were *C. annuum*, 9 were *C. frutescens*, and only 1 was *C. baccatum.* In Ethiopia, despite pepper being economically important, and wide cultivation, its productivity is far below the crop′s potential. According to FAOSTAT [21], world average production of dry pepper is 3.8 t/ha. In Ethiopia, average dry hot pepper production is 1.7 t/ha [9]. In Ethiopia, from the 2010 to the 2018 main cropping seasons, the productivity of red hot pepper dramatically decreased from 2.52 to 1.73 t/ha [26]. In addition, the productivity of red hot pepper declined from 1.73 t/ha in the 2018 to 1.58 t/ha in the 2022 main cropping season [20]. This productivity reduction could be attributed to biotic and abiotic factors such as use of unimproved varieties, lack of good agronomic practices, high temperatures, and pests [15].

## 3. Ecology of Fusarium Wilt Pathogen and Its Disease Symptoms

The fungal genus *Fusarium* comprised multiple plant pathogenic species, which are some of the most harmful phytopathogens worldwide, causing diseases in numerous agricultural crops [27]. *Fusarium* species are cosmopolitan soil phytopathogens, and they survive long periods of time in the soil and cause serious plant diseases. The pathogen′s broad distribution has been attributed to its ability to develop on different substrates and plant species, and to produce spores (chlamydospores) that enable efficient propagation [28]. *Fusarium* species vary in reproduction strategies, and they produce sexual spores as well as three types of asexual spores: (i) microconidia, which are typically produced under all environmental conditions; (ii) macroconidia, which are often found on the surface of diseased plants; and (iii) chlamydospores, which are thick‐walled and produced from macroconidia or older mycelium. The fungus survives for over a decade as a chlamydospore in the soil and infected plant debris [29]. More than 80% of *Fusarium* spp. propagate using asexual spores, but not all of them produce all three types of spores [30].

*F. oxysporum* is the fifth most economically important fungal phytopathogen [31], and it causes great losses on susceptible varieties when soil and air temperature are higher [32]. It is reported that mycotoxins play a key role in the pathogenesis and the aggressiveness of *Fusarium* spp. [33, 34]. The infection cycle of *F. oxysporum* begins when mycelia, germinating asexual conidia or chlamydospore, enter the healthy plant through the root tip, lateral roots, or root wounds. The fungus progresses intracellularly, entering the xylem sap flow and being transported to the aerial parts of the plant, where it forms infection structures. The infection structures that form close to the vascular vessels disrupt nutrient translocation, leading to stomata closure, leaf wilting, and plant death [35, 36]. The most common symptoms of disease are damping‐off, partial wilting, yellowing of older leaves, stunting, complete wilting, and death of plants [36, 37]. Internal vascular tissues of the stem and roots exhibit a characteristic of dark brown discoloration. *Fusarium* species capable of infecting a wide range of plants are classified into different formae speciales, based on the host plant they can infect [38]. Currently, there are 106 well‐described *F. oxysporum* f. sp. [39] and 12 well‐described *F. solani* f. sp. [40].

## 4. Distribution and Intensity of Pepper Fusarium Wilt Pathogen in Ethiopia

**Figure 2:**
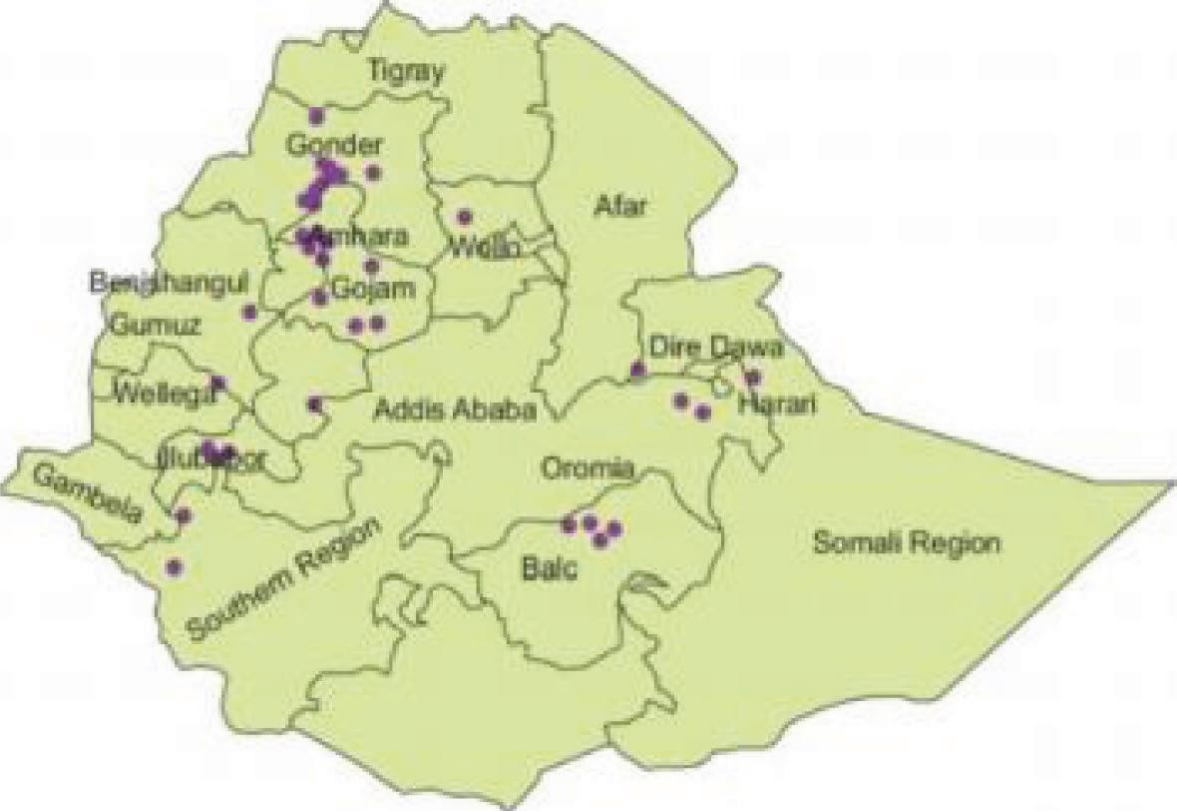
Hot pepper‐producing areas in Ethiopia. Dots represent major producing areas in Amhara, Oromia, SNNP, and Benishangul Regions. Source: Woubit et al. [41].

Currently, hot pepper Fusarium wilt is a big agenda for researchers, producers at different levels, and farmers with long experience in pepper production. According to the FAOSTAT [21], the world average production of dry pepper is 3.8 t/ha. In Ethiopia, average dry pepper production is 1.7 t/ha [9]. Lower productivity is largely due to widespread soil acidity, low organic content, poor nutrients, and pathogenic agents, including fungal Fusarium wilt disease. In Ethiopia, pepper Fusarium wilt distribution and intensity show greater variability among producing districts and regions (Figure 2). Regarding the variation of hot pepper Fusarium wilt, higher incidence in Alaba (91%), Meskan (79%), Mareko (64%), Dugda (50%), Adama (50%), and Adami Tullu (19%) districts have been recorded in the Central Rift Valley of Ethiopia [14]. In NW Ethiopia, major pepper‐growing zones such as W. Gojam, E. Gojam, S. Gondar, W. Gondar, and Awi have consistently reported high levels of Fusarium wilt disease and severe consequences, as the pathogen has become established due to continuous cultivation and a monocropping system. Wilt disease, which causes enormous yield losses, is presently a main constraint of hot pepper production in the W. Gojam zone each year. Alehegn et al. [17] reported variation in pepper Fusarium wilt disease intensity in NW Ethiopia, with severity of 70% recorded in Jabi Tehena, 60% in Hulet Ej Enese, and 57% in Dembecha district. According to Tilahun et al. [15], incidence and severity of pepper Fusarium wilt disease showed variability across NW Ethiopia districts. The average disease severity of 37%, 47%, 50%, and 53%, respectively, was recorded in Womberma, Jabi Tehinan, Dembecha Zuria, and Burie Zuria districts. In the Assosa and Kamashi Zones, it reached up to 50% [42]. According to Fulya Baysal‐Gurel and Kabir [19], wilt‐inducing pathogens such as *Rhizoctonia* spp., *Fusarium* spp., *Verticillium* spp., and *Phytophthora* spp. can result in 50%–75% yield losses in crops like vegetables and fruits. In Ethiopia, wilt‐causing fungal pathogens were reported to be the leading problem of pepper crops, causing 86% [18]. Research results indicated that the pepper Fusarium wilt pathogen has shown a high level of variation in disease severity across the country; the variation could be attributed to agroecological differences, agronomic practice, variety used, cropping system, and disease management methods. The growth of *Fusarium* spp. are largely determined by abiotic conditions, temperature, and humidity [32]. Soil types, cropping system, agricultural practice may influence the diversity of *Fusarium* in soils [43, 44].

## 5. Factors Attributing to Pepper Fusarium Wilt Disease Distribution in Ethiopia

In Ethiopia, hot pepper production practice is characterized by rain‐fed cultivation, use of local variety, planting on acidic soil (pH 5–6.5), and continuous cultivation with a monocropping production system. These agronomic practices favor Fusarium wilt disease and epidemic development. Climatic conditions, notably temperature and precipitation, strongly affect the incidence of *Fusarium* diseases [45]. Research results indicated that *Fusarium* thrives in acidic soil, which is common in Ethiopia. According to Assefa et al. [46], high temperature, moisture, and soil type are found to play a crucial role in Fusarium wilt development. This implies that significant Fusarium wilt disease intensity variation occurs within the major pepper‐producing regions in the country. Research results indicated that there have been variations in pepper Fusarium wilt disease intensity across and within pepper‐producing districts in each region [14, 15, 17]. Variation in pepper Fusarium wilt diseases across and within pepper‐producing districts is attributed to various factors, including sources of seed, cropping system, soil types, altitude, agronomic practices, climatic conditions, and disease management methods. According to Gabrekiristos et al. [14], most farmers in the central rift valley areas obtained seeds stored from the previous season and/purchased from the local market that showed the highest incidence of Fusarium wilt disease. Alehegn et al. [17] reported that fields inspected at an altitude of < 2000 masl showed comparably higher (73%) mean pepper Fusarium wilt disease incidence and severity (70%) than fields located at an altitude of ≥ 2000 masl, with disease incidence of 30% and severity of 27%. Moreover, frequent weeding reduced disease incidence by 42% and severity by 41% as compared with less frequent weeding (1–3) fields across the surveyed districts. The author also indicated that soil type influenced wilt pressure in the studied areas. Pepper farms characterized by the Nitisol soil type lowered disease incidence by 24% and severity by 27% compared with the Vertisol type. The majority of pepper growers (84%) in Ethiopia practiced sole cropping systems, which resulted in higher disease intensities than intercropping and mixed cropping systems [17]. Generally, planting pepper in a sole cropping system with a dense plant population (≥ 20 plants per m^2^), and without wilt management had the highest disease incidence and severity. Research results indicated that soil characteristics, cropping systems, agricultural practices, and other activities may influence the diversity of *Fusarium* in soils [43, 44].

## 6. Hot Pepper Fusarium Wilt Disease Management Strategy

### 6.1. Cultural Practices

Indeed, crop rotation, tillage, and addition of organic amendments may provide some control of soil‐borne pathogens through different microbial direct and indirect mechanisms [47]. A 4‐ to 6‐year rotation with nonhost crops (ex. cereals, grasses, and brassica) can reduce inoculum density. Crop rotation may reduce the severity and incidence of diseases caused by *Fusarium* spp. [48]. For example, compared with the tomato monoculture, soil management under wheat–tomato rotation changes soil microbial composition by increasing the abundance of microbial taxa such as *Bacillus*, *Paenibacillus*, *Pseudomonas*, *Streptomyces*, *Aspergillus*, *Penicillium*, and *Mortierella*, which may control Fusarium wilt of tomato [49]. Improving soil drainage, using a raised bed, and adjusting soil pH can create less favorable conditions for the pathogen. Covering moist soil with transparent plastic sheets during hot months can reduce soil pathogenic inoculum through solar heating. Effectiveness depends on the prevailing climate conditions. Removing and destroying infected plant debris, disinfecting tools, and using clean irrigation water to prevent pathogen buildup and spread.

### 6.2. Organic Soil Amendment Method

Long‐term intensive agriculture has resulted in soil nutrient depletion and salinity accumulation, thus significantly reducing soil productivity. In Ethiopia, pepper productivity is lower; this is mainly due to widespread soil acidity, low organic content, poor soil nutrients, and pathogenic agents, including fungal Fusarium wilt disease. Soil suppressive to *Fusarium* diseases has been documented in different regions of the world. Suppressive soil contains biocontrol microorganisms, which act by inducing plants′ resistance to the pathogen, competing with or inhibiting, or parasitizing the pathogen [50]. Organic soil amendment is one important soil suppressive practice, as the incorporation of compost, green manure, biochar, or biofumigant crops can enhance soil nutrients, microbial diversity, and suppress the Fusarium wilt pathogen. Organic amendments represent a favorable environment for beneficial microorganisms that are able to combat phytopathogenic *Fusarium* species [51]. Soil amendment is an alternative choice to manage bacterial wilt and root rot diseases in the major solanaceous crop‐growing regions of Ethiopia [52, 53]. *Fusarium* thrives in acidic soil, which is common in Ethiopia. Organic soil amendment has a high liming potential, raising the soil pH to the level where the fungus struggles to survive. According to Reijs et al. [54], Fusarium wilt disease incidence is reduced by up to 80% when organic matter is applied to the soil. Soils supplemented with coffee residue compost or rapeseed meal exhibited suppressiveness to *F.* oxysporum mediated wilt [55]. Addition of vermicompost reduced tomato infection by *F. oxysporum* f. sp. *lycopersici* [56]. According to Jafri et al. [57], fern (*Dryopteris nigropaleacea*) used as soil amendment against *F. oxysporum* f. sp. *lycopersici* significantly enhanced fresh tomato yield (31%) and reduced disease incidence by 46% and severity by 61%.

Biochar soil amendment can improve physical, chemical, and biological characteristics of soil and boost nutrient availability [58, 59]. Biochar soil amendment promotes mycorrhizal fungi [60], enhances growth‐promoting rhizobacteria, N2‐fixing bacteria [61], *Trichoderma* spp. [62], alters soil microbial populations and functions [63], improves root‐associated bacterial community [62, 64], and induces systemic plant disease resistance [65]. According to Elad et al. [65], biochar‐treated soil induced systemic resistance to tomato gray mold and pepper powdery mildew pathogens. Lu et al. [63] reported biochar amended soil significantly reduced tomato bacterial wilt disease and increased soil microbial population density and activity. Similarly, biochar and compost exhibit a great potential in suppressing *Fusarium* chlamydospore infection in tomato [66]. Biochar applied at 2% promoted tomato growth and decreased Fusarium wilt severity [67]. Samuel et al. [68] found the application of vermicompost and biochar reduces root rot disease and improves bean productivity. Rogovska et al. [69] showed biochar‐treated soil significantly reduced soya bean root rot (*Fusarium virguliforme*) severity. Asif et al. [70] found that biochar soil treatment significantly decreased colonization and survival, and inhibited Fusarium wilt of cotton. Generally, biochar soil amendment disease suppression mechanisms are attributed to (i) induction of systemic resistance in host plants, (ii) enhanced abundance and activities of beneficial microbes, (iii) modification of soil quality in terms of nutrient availability and liming effect, (iv) direct fungitoxic effect, and (v) sorption of allelopathic and phytotoxic compounds [61, 62]. Despite multiple benefits of organic soil amendment in Ethiopia, its application is very rare.

### 6.3. Biological Method

Plant‐beneficial microorganisms present in the rhizosphere may protect plants from *Fusarium* pathogens through different modes of action, including (i) induction of resistance in the plant, (ii) competition for space and nutrients, (iii) production of different metabolites or lytic enzymes, or (iv) parasitism [71]. Some of them are also able to inhibit mycotoxin synthesis or to enhance their detoxification [71, 72]. Certain biocontrol microorganisms have multiple modes of action, which may be expressed simultaneously or sequentially [72].

Trichoderma species are well‐known as model organisms due to their ability to multiply, spread, isolate, and culture easily [73]. The use of *Trichoderma* for Fusarium wilt biocontrol is not only safe for farmers and consumers, but it is also an environmentally friendly option. *Trichoderma* employs several biocontrol mechanisms to inhibit the growth of a variety of phytopathogenic organisms, including bacteria, nematodes, and fungi (*Fusarium*, *Pythium*, *Botrytis*, and *Rhizoctonia*), either directly (e.g., hyperparasitism, competition, and antibiosis) or indirectly by increasing stress tolerance, promoting plant growth, and producing several secondary metabolites, enzymes and pathogenesis‐related proteins. Several studies have shown that *T. harzianum* is an effective fungal bioagent against *F. oxysporum* f. sp. *capsici* [74, 75]. A recent research finding indicated that *Fusarium*‐infected soil inoculated with *Trichoderma* spp. resulted in a significant *F. oxysporun* f. sp. *ciceris* disease reduction (80%–88% DI) and increased grain yield (65%) per ha in chickpea [76]. According to Abayeneh [74], of 32 in vitro tested *Trichoderma* isolates, six (*T. harzianum* [TD1], *T. asperellum* [TD5], *T. viride* [TD7], *T. hamatum* [TD11], *T. virens* [TD15], and *T. longibrachiatum* [TD21]) strains showed (46%–94%) biocontrol activity toward the tested pathogen. The three (TD5, TD1, and TD7) strains showed effective antagonists against *F. oxysporum* f. sp. *capsici* of pepper with a colonization percentage of 89.5%, 90%, and 94%, respectively. More than eight *Trichoderma* spp. were molecularly identified and maintained in the Ethiopian Ambo Agricultural Research Center. These *Trichoderma* spp. have been used in a variety of plant disease management applications nationwide. *T. harzianum*, *T. viride*, and *T. asperellum* have been effective and potential bioagents against *Fusarium* species in diverse environments involving various crops, including *Capsicum* [77].

### 6.4. Chemical Method

Various fungicides, both contact and systemic, have been tested and found to be effective against Fusarium wilt of pepper [78]. Fungicides that are used as seed treatment for seed‐borne diseases eliminate seed‐borne inocula, as well as soil treatment and/or seedling root dipping to suppress soil‐borne pathogens such as Fusarium wilt. Carbendazim (0.2%), carbendazim + mancozeb (0.25%), and tebuconazole + trifloxystrobin (0.1%) soil drenching were found to be effective in eliminating the disease and increasing fruit yield [79]; however, risks include fungicide resistance and cost. Soil fumigants (methyl bromide) are effective but expensive, hazardous, and largely in accessible. Due to the outbreak of Fusarium wilt epidemics, small‐scale farmers and other pepper producers are forced to use only synthetic fungicides to manage Fusarium wilt on *Capsicum* species. However, pesticides to treat plant diseases in general and Fusarium wilt in particular have disadvantages due to pesticide resistance and environmental or soil hazards [80].

### 6.5. IPM

In Ethiopia, the most widely cultivated and demanded pepper varieties such as Bako Local, Mareko Fana, Melka Zala, and Melka Oli are highly susceptible to *F*. *oxysporum*; however, the Melka Shote variety is moderately susceptible to this pathogen [81]. According to Alehegn et al. [82], the Mareko Fana variety grown in control plots showed the highest disease severity (54%) and disease progress rate (0.0114 units/day). Aklilu et al. [83] found that both Melka Awaz and Melka Zala pepper varieties displayed moderate resistance to *F*. *oxysporum.* According to Tadesse et al. [84], of 52 Ethiopian pepper accessions tested for *F*. *oxysporum* under greenhouse conditions, only two accessions (225733 and 25828) showed resistance reactions.

Managing Fusarium wilt is challenging, and deploying a single technique is inefficient due to the pathogen is cosmopolitan, broad host range, genetic diversity, prolonged survival in the soil and survival on vegetation as a latent infection [85]. Therefore, it is imperative to integrate various wilt disease management strategies that are less reliant on pesticides and less destructive to soil, water, and biodiversity [14]. The country must develop integrated Fusarium wilt management method that consider specific agroecology of pepper‐producing areas. According to Mohammed et al. [86], integrated management with cultivars, *T. harzianum*, and metalaxyl‐M + mancozeb effectively manages Fusarium wilt on *Capsicum* species. The combined approach led to a 75.3% reduction of Fusarium wilt on the Bako Local cultivar and 80% on the Melka Zala hybrid variety during the 2021 pepper growing season. According to Alehegn et al. [82], a combination of compost, host resistance, and chemical treatments on pepper seeds and seedlings effectively managed pepper Fusarium wilt disease in Ethiopia. Melka Zala variety treated with Apron Star 42% WP and transplanted into compost‐treated plots exhibited the lowest disease severity (23%) and disease progress rate (0.0034 units/day), and improved yield compared with the Mareko Fana variety [82].

## 7. Conclusion

Fusarium wilt is a major pepper‐production constraint and the most frequently occurring pathogen isolated from diseased pepper in Ethiopia [15, 82]. The use of local variety and continuous cultivation with monocropping system favor Fusarium wilt pathogen and epidemic development in the country.in Ethiopia, wilt‐causing fungal pathogens were reported causing pepper 86% yield loss [18]. Managing Fusarium wilt is challenging due to the pathogen′s broad host range, genetic diversity, and prolonged survival in the soil [85]. Pepper Fusarium wilt management via cultural methods is cost effective and ecological sustainable but it is labor demanding, and less effective at the early stage. The use of *Trichoderma* as a biological control method for Fusarium wilt is safe and eco‐friendly; however, its wide use is limited as biological agent requires large‐scale formulation and commercialization [86]. Organic soil amendment is important soil suppressive practice; it can enhance microbial diversity, and suppress Fusarium wilt pathogen. However, its application in Ethiopia is at the infant stage. No single method provides a complete control to Fusarium wilt pathogen, and its management requires a shift from reliance on a single solution to a holistic disease management strategy. Integrating host resistance, cultural practices, organic soil amendment, and biological control form the cornerstone of sustainable management. In Ethiopia, most pepper Fusarium wilt research focused on reporting the disease incidence and severity. However, it is essential to investigate the factors attributed to Fusarium wilt distribution and epidemic development and also focus on screening and identification of pepper Fusarium wilt resistance genotype from locally available accessions, and determine integrated effects of host resistance and organic soil amendment as alternative Fusarium wilt management strategy.

## Funding

No funding was received for this manuscript.

## Conflicts of Interest

The author declares no conflicts of interest.

## Data Availability

The data that support the findings of this study are available from the corresponding author upon reasonable request.
